# On the Value of Using 3D Shape and Electrostatic Similarities in Deep Generative Methods

**DOI:** 10.1021/acs.jcim.1c01535

**Published:** 2022-03-10

**Authors:** Giovanni Bolcato, Esther Heid, Jonas Boström

**Affiliations:** † Molecular Modeling Section, University of Padova, 35131 Padova, Italy; ‡ Department of Chemical Engineering, Massachusetts Institute of Technology, Cambridge, 02139 Massachusetts, United States; § Medicinal Chemistry, Early CVRM, BioPharmaceuticals R&D, AstraZeneca, 431 50 Mölndal, Sweden

## Abstract

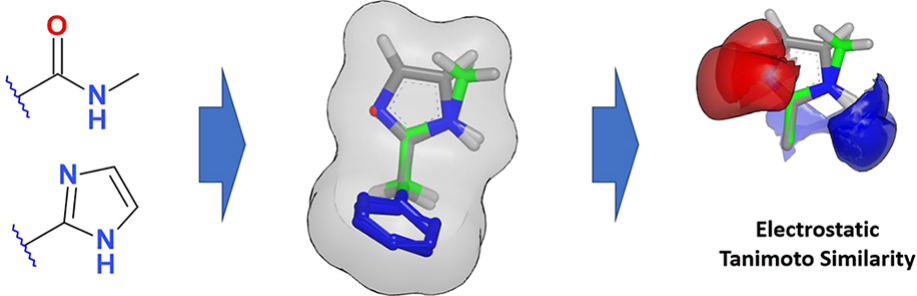

Multiparameter optimization, the heart of drug design, is still an open challenge. Thus, improved methods for automated compound design with multiple controlled properties are desired. Here, we present a significant extension to our previously described fragment-based reinforcement learning method (DeepFMPO) for the generation of novel molecules with optimal properties. As before, the generative process outputs optimized molecules similar to the input structures, now with the improved feature of replacing parts of these molecules with fragments of similar three-dimensional (3D) shape and electrostatics. We developed and benchmarked a new python package, ESP-Sim, for the comparison of the electrostatic potential and the molecular shape, allowing the calculation of high-quality partial charges (e.g., RESP with B3LYP/6-31G**) obtained using the quantum chemistry program Psi4. By performing comparisons of 3D fragments, we can simulate 3D properties while overcoming the notoriously difficult step of accurately describing bioactive conformations. The new improved generative (DeepFMPO v3D) method is demonstrated with a scaffold-hopping exercise identifying CDK2 bioisosteres. The code is open-source and freely available.

## Introduction

A crucial task in all drug discovery projects is designing molecules against multiple, often contradictory objectives.^1^ Much of today’s drug hunters’ time is therefore spent on attempting to find an optimal compromise where all desirable properties are satisfied in a single molecule. The use of sophisticated computational methods, leveraging high-quality data sets to help solve this task, is thus conceptually very attractive.

Recent advances in artificial intelligence (AI) and machine learning (ML) have given rise to an immense popularity of inverse design,^2^ and the field shows little signs of slowing down.^3^ In inverse design, desired properties are specified *a priori*, and such methods generate compounds fitting that description.^4^ Significant progress has been made in this area, and a plethora of approaches for deep learning in molecular design has been published in the last few years.^2^ Many methods include reinforcement learning^5−7^ to generate molecules, most often in the form of SMILES strings.^8^ Other popular methods include generative methods such as recursive neural networks, generative adversarial networks, or variational autoencoders, which are sometimes steered with reinforcement learning to control the molecular properties. The SMILES format in itself is nothing but amazing.^9^ Using SMILES is also convenient for the AI algorithm since it is trivial to manipulate and transform a string. In addition, there are success stories of using SMILES in the area of generative design.^10^ However, all molecules are 3D objects, and a conservative modification to a SMILES string may cause a large effect in their 3D structure. Examples include the removal of brackets denoting substitution, such that a Y-shaped compound becomes linear, or the removal or changing of ring-closing locants. Therefore, optimization of molecular structures cannot be smooth in the space of 3D properties even though the SMILES strings change by only small amounts from iteration to iteration of the AI algorithm. We have previously presented a fragment-based generative approach (DeepFMPO) that addressed these modifications to the structure issue, albeit as 2D descriptions.^11^ Here, we introduce a significant extension to DeepFMPO, using detailed descriptions of 3D properties to represent molecules more accurately.

The shape and electrostatic properties of molecules are primary determinants of molecular recognition and should consequently be the method of choice when comparing the similarity of molecules encountered at various stages in drug design. Even though these methods have been used to achieve major impacts in related areas (e.g., virtual screening leading to the discovery of novel and unexpected chemotypes^12−14^), they have been largely unexplored in the context of *de novo* generative methods, although promising attempts have been made.^15,16^ One reason for the reluctance of using 3D methods is the challenge of obtaining accurate descriptions of molecules’ bioactive conformations. In this work, we reduce this notoriously difficult step by using 3D fragments of the complete target compounds.

Moreover, much of the work in the generative *de novo* design area has been focused on the development of maximally expressive methods whose purpose is to explore the entire chemical space. Our approach is different in this regard since it specifically rewards the generation of molecules that are similar to known lead compounds. Another such method is the recently published MolDQN method, which maximizes a “drug-likeness” (QED) score while also maintaining similarity to the original molecule.^17^ Virtual screening is a related approach to generative methods^18^ and can also be a powerful method for finding hits and lead compounds with desired properties. However, a virtual screen is limited in regard to what is in the queried databases (i.e., it is not possible to find something that is not there).

Here, we describe a new open-source python package, ESP-Sim, for calculating shape and electrostatic similarities, and its implementation in DeepFMPO.^11^ We highlight its usefulness with a scaffold-hopping study that identifies bioisosteres for a set of CDK2 kinase inhibitors.^20^

## Methods

**Figure 1:**
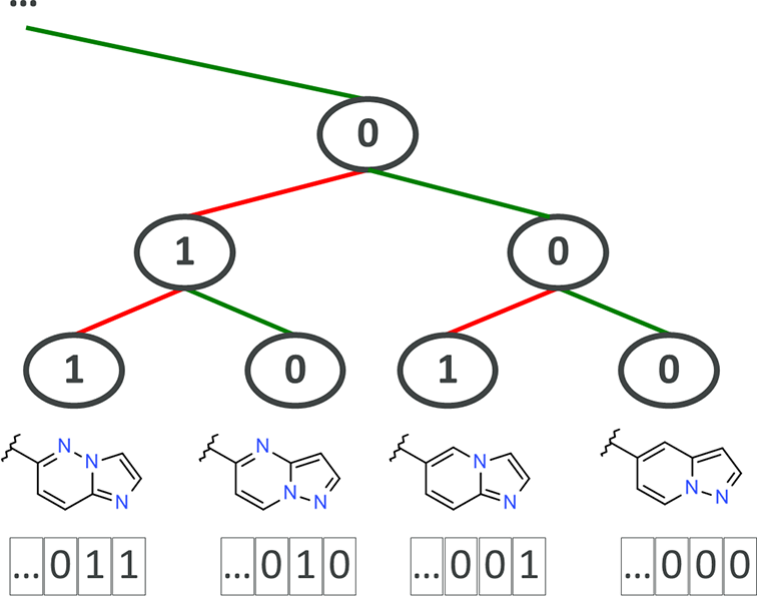
Snippet of the balance binary tree used in DeepFMPO. Fragments that are similar are placed close to each other. The encoding of a fragment is determined by the path from the root to the leaf. Every branching to the left adds a “1” to the end of the encoding, and a branching to the right adds a “0”.

The DeepFMPO method is based on an actor–critic model for reinforcement learning.^11^ It is a fragment-based generative method that learns how to modify compounds and improve them. That is, molecules are split into fragments, and these fragments are replaced with other similar fragments in the (deep) learning process of generating novel molecules with optimal properties. Technically, the fragments are encoded into binary strings, and similar fragments are assigned with similar encodings. This is achieved by constructing a balanced binary tree. In the process of assembling the tree, similarities between all fragments are calculated. Fragments are paired in a greedy bottom-up manner, where the two most similar fragments are paired first. The joining procedure is repeated until all fragments are put together in a single tree. Subsequently, this information is used to generate encodings for all fragments. The paths from the root to the leaves define the encoding for each fragment. For every branch in the tree, a one (“1”) is added to the encoding when going to the left, and a zero (“0”) is added when going to the right, see Figure 1. The rightmost character in the encoding corresponds to the branching closest to the fragment. In this process, the pairwise similarity between all fragments is calculated. There are many ways to calculate chemical similarities, and the most used approaches currently employ 2D fingerprints.

**Figure 2:**
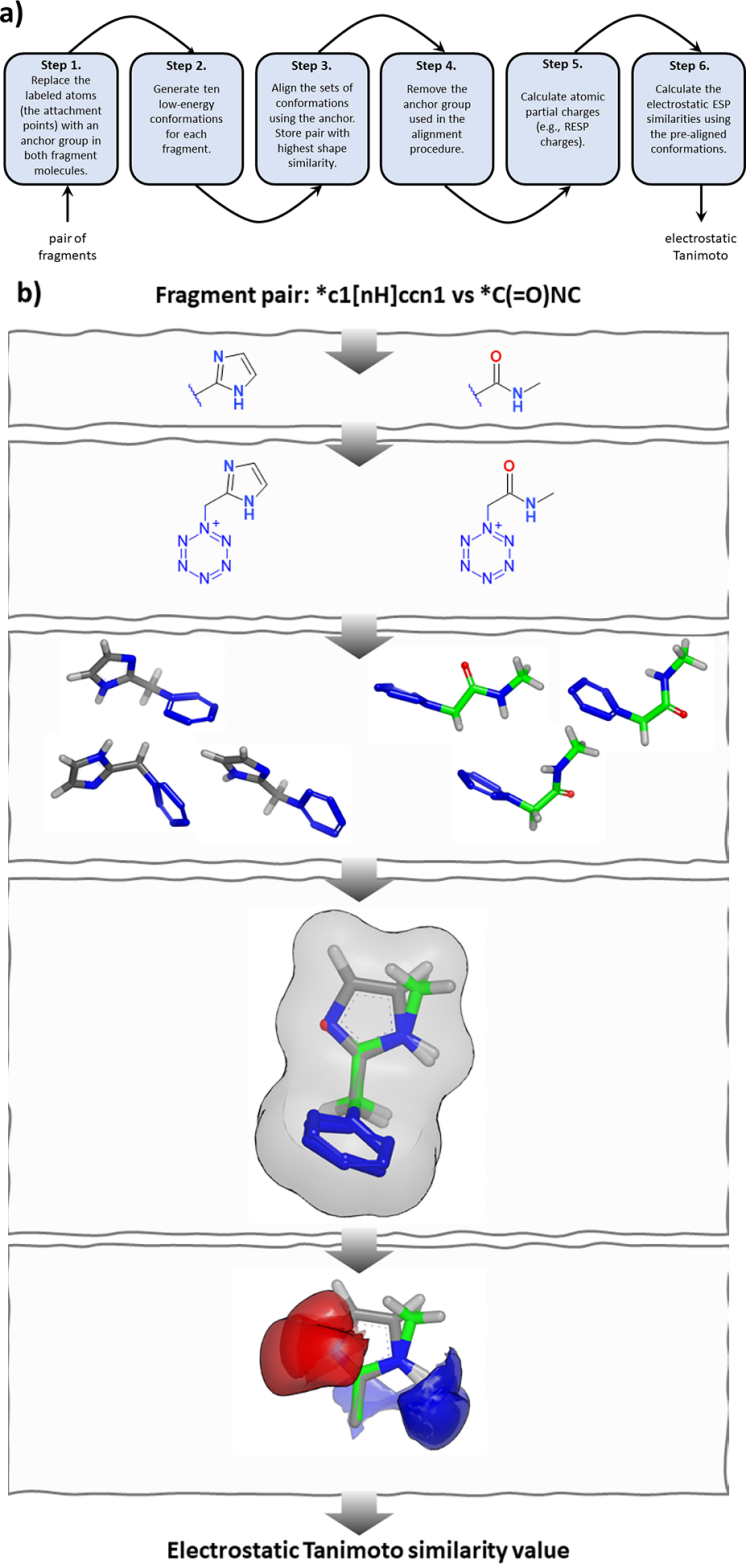
(a) Stepwise procedure to obtain the electrostatic shape potential similarity values for pairs of fragments. (b) Example of the corresponding procedure in graphics.

Here, we present a new implementation of DeepFMPO utilizing a 3D-based molecular alignment method, where the electrostatic potential (ESP) similarity between pairs of fragments is calculated. To this aim, we developed an open-source python package, ESP-Sim, which calculates the overlap integrals of the electrostatic potentials (generated from Coulomb potentials) of two molecules or fragments. Within DeepFMPO, the computation of ESP similarities can be broken down into six steps for each fragment pair (see Figure 2a) and is described in more detail below. Steps 2, 3, and 6 correspond to function calls of the ESP-Sim package, whereas steps 1 and 5 are innate to DeepFMPO. It is worth noting that this fragment alignment approach eliminates the challenging step of generating bioactive conformations for complete molecules as well as alleviates the issue of aligning them correctly.

### The Molecular Alignment of Fragments

All single bonds in a molecule that extend from a ring atom are broken in the DeepFMPO process, creating the molecular fragments. The attachment atoms (previously connected with a single bond) are labeled in this step. To calculate ESP similarities, the fragments must be aligned in 3D. Here, a conformational search is conducted generating an ensemble of low-energy conformers for all fragments containing rotatable bonds, using the ETKDG method^21^ as implemented in RDKit.^22^ As default, a maximum of 10 conformations of each fragment is generated. An anchor group is connected to the fragments’ attachment atom and serves as a template in the alignment procedure. The coordinates of the anchor group are fixed in 3D space. The rationale for this step is that ligands containing related fragments typically bind in a similar orientation,^23,24^ and these fragments will frequently make similar ligand–protein interactions. Consequently, to ensure that the fragments are aligned as accurately as possible, an anchor group is attached to the fragments and used in the molecular alignment step. The anchor group was arbitrarily chosen to be a hexazine ring with a methylene linker subunit. This group is of reasonable size for a template and highly unique (i.e., hexazines are never present in drug-like molecules) for easy identification and removal downstream in the process. A few experiments were conducted with other types of structural fragments as anchors to gauge possible conformational effects (*vide infra*). For each pair of fragments, the pair of conformations with the best shape overlay in terms of the highest shape Tanimoto value is stored. The anchor is then replaced with a hydrogen (see Figure 2). In cases where fragments include several labeled atoms, they are replaced with a methyl group. In this manner, all labeled atoms are replaced by a methyl, which may be considered neutral in terms of electrostatic similarities. Finally, the ESP Tanimoto value is calculated between the pair of conformers with the best shape alignment (see the Electrostatic Similarity Calculations section below).

### Electrostatic Similarity Calculations

The presented ESP-Sim method uses the cheminformatics toolkit RDKit^22^ to generate different conformations of a molecule with (or without) a constrained anchor or core group and computes shape and electrostatic potential similarities between pairs of conformers. Alternatively, ESP similarities can be computed on prealigned molecules. The electrostatic potential similarity is computed via the overlap integral of the Coulomb potentials of two molecules, as well as their respective self-overlap integrals as either Tanimoto^25,26^ or Carbo similarity.^27,28^ The Coulomb potential *V*(***r***) describes the electric potential at a point ***r*** as a sum of potentials of point charges *q_i_* at points ***r***_*i*_ as
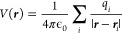
where ϵ_0_ is the vacuum permittivity. Since analytic integration of the Coulomb potential at ***r*** = 0 is not possible, we provide options to either approximate each potential with a sum of three Gaussian functions and integrate the fit function analytically analogous to Good *et al.*(29) or to perform a Monte Carlo integration over the space outside of the van der Waals radii of each atom and inside a user-defined margin. Partial charges can either be supplied by the user, calculated via RDKit (Gasteiger or MMFF94 charges), predicted via a recent machine learning (ML) model,^30^ or computed using the open-source quantum chemistry program Psi4,^31^ with the option of using restrained electrostatic potential (RESP) charges.^32^ There are a range of different methods and basis sets available in Psi4, for example, the often recommended combination of using the B3LYP method and the 6-31G** basis set, although using them can be computationally demanding. Within DeepFMPO, Dask,^33^ a library for parallel computing in Python, is used to speed up the process. It should be noted that the RESP/Psi4 method is not parametrized for atoms beyond the atomic number of argon. To allow for larger atoms (e.g., bromine), their van der Waals (vdW) radius needs to be specified separately. In this code version, we set the vdW radii for bromo to 1.8 (file: resp/vdw_surface.py), following the GAMESS scheme^34^ derived from the Merz–Kollman–Singh publication.^35^ In addition to electrostatic similarities, ESP-Sim can furthermore output the shape Tanimoto similarity of molecules, describing the volume overlap. For DeepFMPO, we used the Tanimoto similarity of electrostatic potentials obtained via fitting to Gaussian functions (ESP-Tanimoto). We furthermore provide an option to add the volumetric shape score resulting in an ESP-TanimotoCombo score.

## Results

In the following, we showcase the performance of ESP-Sim on a variety of benchmark tasks. We then perform a retrospective case study, where we aim to demonstrate the value of using shape and electrostatic similarities in scaffold-hopping exercises. Scaffold hopping is a method for identifying bioisosteric replacements^36,37^ with the intention of retaining biological activity of analog compounds but also improving other relevant molecular properties. It can also be used as a design strategy for intellectual property (IP) reasons.

### ESP-Sim Benchmark Studies

To evaluate the influence of the employed partial charge distribution on the observed scores within ESP-Sim, ESP similarities were computed for the same molecule at the same geometry but with different partial charges. As ground truth, quantum mechanically (QM) obtained RESP charges at a high level of theory (MP2 with a polarizable PVTZ basis set) were used,^30^ on a selection of about 3000 neutral molecules. RESP charges are specifically designed to reproduce the electrostatic potential of a molecule so that a comparison of electrostatic potentials obtained from different charge distributions to the QM RESP charges allows for a detailed assessment of the quality of each approach for similarity comparisons. We evaluated Gasteiger^38^ (default in RDKit), MMFF94,^39^ and AM1-BCC^40,41^ partial charges, as well as a machine learning model (ML).^42^ The ML partial charge model is provided with the ESP-Sim package on Github. Table 1 provides an overview of the observed mean absolute deviations of the respective partial charges from the RESP charges, as well as the ESP similarities evaluated via Carbo or Tanimoto similarities. We find that AM1-BCC charges reproduce the QM electrostatic potentials best followed by the deep learning model, MMFF, and Gasteiger.

**Table 1:** Mean Absolute Deviations between Gasteiger, MMFF, ML, or AM1-BCC Partial Charges *q* Compared to RESP Charges, as well as Similarities of Electrostatic Potentials Compared to RESP Evaluated Either via Carbo or Tanimoto Similarity

partial charges	MAE *q* [e]	ESP-Sim (Carbo)	ESP-Sim (Tanimoto)
Gasteiger	0.16	0.78	0.60
MMFF	0.17	0.80	0.64
ML	0.17	0.85	0.61
AM1-BCC	0.12	0.88	0.78

**Figure 3:**
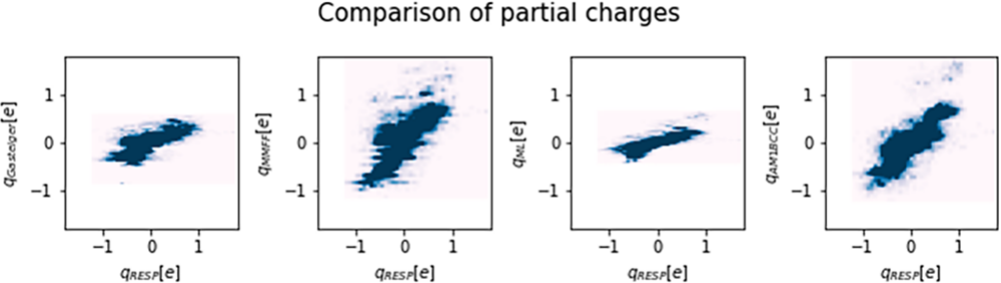
Heat maps of quantum mechanical RESP partial charges compared to Gasteiger, MMFF, ML, or AM1-BCC partial charges.

**Figure 4:**
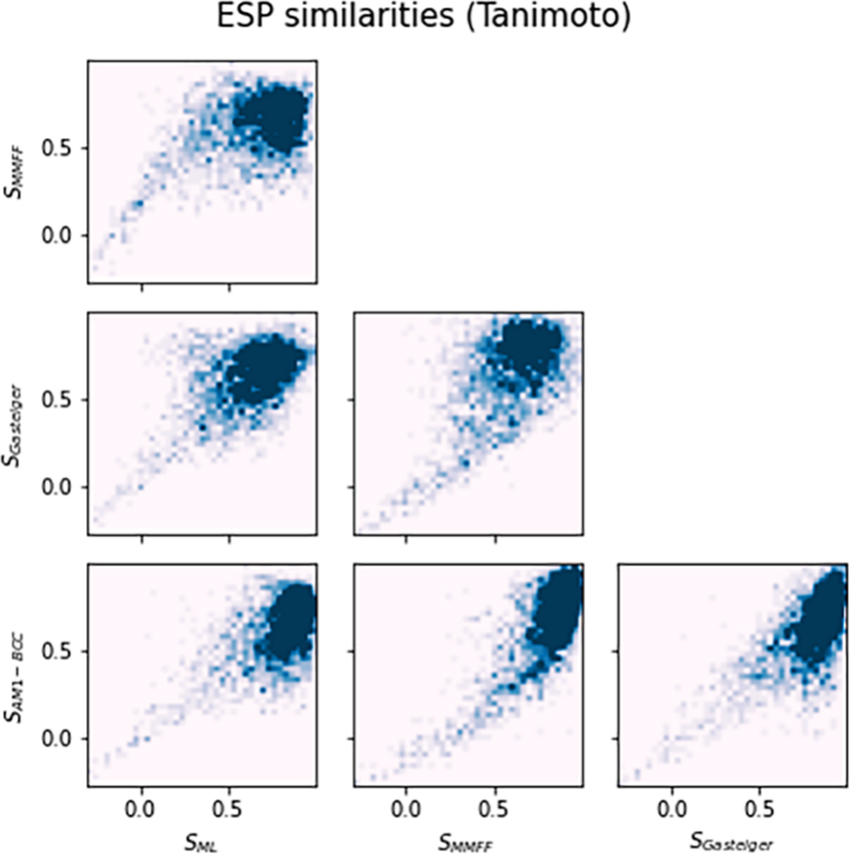
Electrostatic potential similarities between molecules with RESP partial charges to molecules with Gasteiger, MMFF, ML, or AM1-BCC partial charges (at the exact same geometries evaluated via Tanimoto similarity). An analogous figure for Carbo similarity is given in the Supporting Information.

Figure 3 depicts heat maps of the QM atomic charges compared to Gasteiger, MMFF, ML, and AM1-BCC charges. Although there is no perfect correspondence of QM charges to any of the evaluated charges, we find the highest agreements for AM1-BCC charges. Notably, Gasteiger and ML charges lead to narrower ranges than MMFF and AM1-BCC charges. This is also reflected in the Carbo and Tanimoto ESP similarities in Table 1. The Carbo metric, which is largely insensitive toward the magnitude of a function,^43^ yields more favorable scores for ML and AM1-BCC, in contrast to the Tanimoto metric, which is more sensitive to the absolute magnitudes, thus ranking MMFF better than ML. However, these differences are small, and a comparison of observed similarities via different charge distributions in reference to QM RESP shows similar trends between all options (Figure 4). We can therefore assume that even Gasteiger charges lead to a fair depiction of the electrostatic potential for most of the molecules. In fact, benchmarking of ESP-Sim on protein-docking databases shows little dependence of ranking metrics on the employed partial charge distribution, as detailed in the following.

We furthermore compared ESP-Sim scores to similarities obtained via the state-of-the-art tool EON^44^ for about 450 fragments generated by DeepFMPO for various partial charge distributions. We find a strong correlation, with a Spearman correlation coefficient of about 0.8. A detailed analysis is given in the Supporting Information. In addition, we assessed the ability of ESP-Sim scores to identify potential ligands to protein targets. We compared the performance of ESP-Sim electrostatic and shape similarities to a set of rescoring functions^45−51^ on the dopamine D4 receptor, for which experimental data on active and inactive compounds is known.^52^ We furthermore benchmarked ESP-Sim on the 102 DUD-E targets^53^ and compared its performance against a variety of ligand-based approaches.^54−59^ For both comparisons, we find that ESP-Sim electrostatic and shape similarities perform very well. Details on these benchmarks are given in the Supporting Information.

### Assessing Various Molecular Similarity Measures

**Figure 5:**
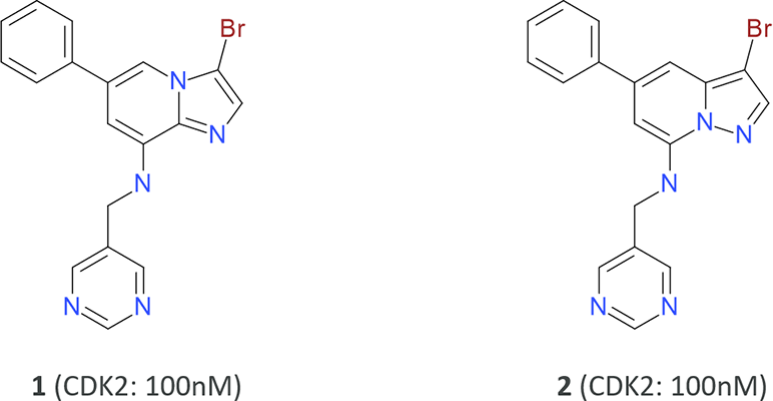
Two equipotent CDK2 kinase inhibitors. CDK2 inhibitors containing the related bicyclic heterocycles imidazopyridine (**1**) and pyrazolopyridine (**2**) were discovered through high-throughput screening by Fischmann *et al.*(20) and here used as a scaffold-hopping example.

A frequently occurring scenario is that a drug hunting team has identified a promising compound, from an internal lead generation effort or from the literature, that needs optimization. For the sake of argument, compound **1**(20) in Figure 5 is such a compound.

With compound **1** at hand, the design question is then “which compound should we make next?”. The optimization task usually includes improving molecular properties (e.g., permeability, solubility, clearance, selectivity, etc.) and perhaps also IP-related issues. A common scenario then is for the project team to try to come up with ideas of novel central rings to be introduced as scaffold replacements. In this context, it should be noted that heterocyclic rings are often considered special and typically end up in different patent applications.^60^ Also, with regard to calculating molecular properties (e.g., lipophilicity), many 2D-based methods are not adequately parametrized and have difficulties in assessing heterocyclic compounds accurately.^61^ So, how can breakthrough ideas for novel central rings be generated and which methods can be used to do it? Here, compound **2** (Figure 5) is one answer to the question “what to make next?”. It is equipotent to compound **1** and, importantly, contains a different but related central scaffold. That is, the bicyclic heterocycles in compound **1** (imidazo(1,2-*a*)pyridine) and compound **2** (pyrazolo(1,5-*a*)pyridine) are both nine-membered ring systems with identical substituents.

**Figure 6:**
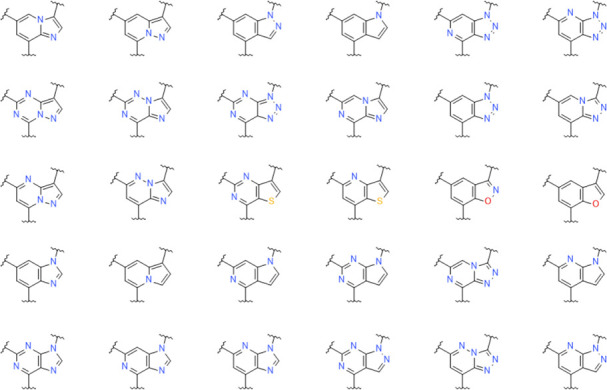
Bicyclic heterocyclic scaffolds in ChEMBL compounds matching the SMARTS pattern “[A][cH0]1[c,n][c,n]([A])[c,n]2[c,n][c,n][c,n]([A])[c,n]2[c,n]1”.

To investigate how different methods predict the similarity of these kinds of central bicyclic heterocyclic scaffolds, we first generate a data set of fragments containing the same framework and similar substitution pattern. Thus, the ChEMBL v28 database^62^ was queried for compounds including a nine-membered bicyclic ring system, with three substituents, using SMARTS matching.^22^ For comparison reasons, the substituents were subsequently removed providing 30 different scaffolds, see Figure 6. In this manner, we identified an extensive list of nine-membered bicyclic heterocyclic scaffolds present in drug-like molecules that could potentially act as replacements for the pyrazolopyrimidine in compound **1**.

**Figure 7:**
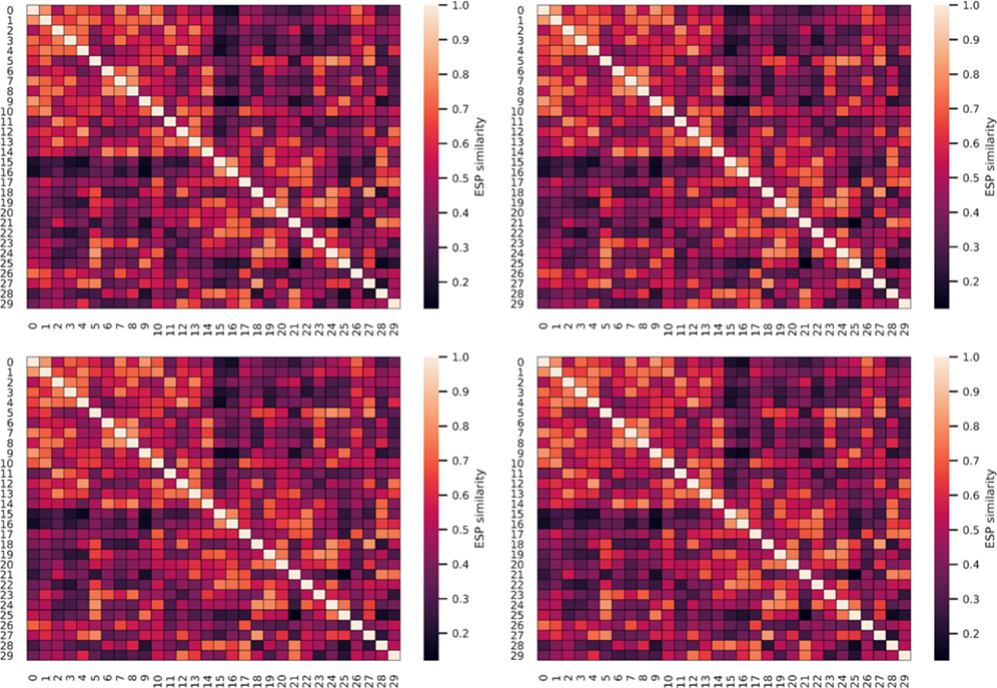
All-against-all comparison experiments were conducted with four structurally different anchor fragments (top left: hexazine, top right: carboxylic acid, bottom left: piperidine, and bottom right: iodine). The different anchors give essentially the same results.

All 30 bicyclic systems were subsequently subjected to pairwise comparisons using a range of standard 2D similarity measures, together with the 3D-based ESP-Sim measure. A summary of the results obtained from each method is reported in Table 2. For completeness, the results using four different anchor fragments (hexazine, carboxylic acid, piperidine, and iodine) are shown in Figure 7. The heat maps are essentially the same, indicating that the method is not dependent on the choice of the anchor fragment.

**Table 2:** Rankings for the **1** vs **2** Fragment Pair, among Pairwise Comparisons of 30 Different Heterocyclic Rings^a^

method	rank (max = 30)
ESP-Sim (B3LYP/6-31G**)	1
Morgan fingerprint (radius 2)	5
Morgan fingerprint (radius 3)	5
MACCS keys’ fingerprints	17
MCS Tanimoto	21
topological fingerprints	22

a The rankings, and Tanimoto values, using a range of different 2D similarity methods available through RDKit and the new ESP-Sim measure are reported. Hexazine was used as an anchor fragment.

The **1** vs **2** fragment pair is top-ranked when using the ESP-Sim (B3LYP/6-31G**) metric but not by the 2D-based methods. The Morgan fingerprints rank the **1** vs **2** pair among the top five (Table 2), which is reasonably high. However, given the challenges and resource investments required to establish new synthetic routes, our experience is that very few alternative ring analogs are explored in real-life projects. Typically, only a couple of ring replacements are made and tested, essentially enforcing that only top-ranked scaffolds would be followed up. Two other observations provide further support for the use of the ESP-Sim method. First, the MACCS keys’ fingerprint resulted in very similar values for many scaffolds (e.g., the Tanimoto similarity values for five scaffolds against the scaffold of compound **1** show identical values of −0.87), suggesting that the MACCS keys’ similarity metric is not sufficient for capturing such subtle differences. Second, there is a couple of clearly structurally dissimilar fragments in Figure 6 (e.g., 1,4,6-trimethylpyrazolo[5,4-*b*]pyridine vs 2,4,7-trimethylimidazo[2,1-*f*][1,2,4]triazine) that are ranked low when using ESP-Sim (as they most probably should) but top-ranked when using Morgan 2D fingerprints.

As a final observation, deriving ESP similarities with methods of lower theory for calculating the underlying partial charges (Gasteiger, MMFF, and HF/3-21G, data not shown) also yielded the **1** vs **2** pair as top-ranked, suggesting that such partial charges may be sufficient and a cost-effective alternative for this data set. We recommend using a higher level of theory, although it is computationally more demanding, for example, RESP partial charges derived using the B3LYP method and the 6-31G** basis set or AM1-BCC partial charges, which were found to reproduce the QM electrostatic potentials best in our benchmark.

### Generating “Sweet Spot” Molecules

Having established the value of using the ESP-Sim measure, the next step was to include it in the generative (DeepFMPO) method. An experiment was set up to mimic a real-world scenario, where a set of lead compounds is optimized toward the sweet spot criteria through a multiparameter optimization process. Three different calculated properties (molecular weight, polar surface area,^22^ and clogP^63^), commonly used in the optimization of leads to candidate drugs, were selected for this purpose. It should be noted that the choice of molecular properties was also selected for practical reasons facilitating reproducibility. Namely, there are methods for calculating them using RDKit.^22^ The aim of the setup was to bias the generation of compounds to fulfill the criteria for the three calculated properties while also maintaining their similarity in shape and electrostatics toward a known set of lead compounds. The agent in the reinforcement learning method was rewarded for producing valid molecules and got a higher reward when generating molecules with properties in the targeted ranges. Since this was a scaffold-hopping exercise, with the goal of identifying a new bioisosteric scaffold, the minimum and maximum target values for the three properties were centered around the corresponding values for compound **1** (i.e., 320 < MW < 420, 2.3 < clogP < 4.3, and 45 < PSA < 65).

The library of input fragments was generated from a set of structurally diverse compounds known to exhibit inhibitory effects against kinase targets, including compounds that have shown activity against the specific biological target of interest (CDK2). The data set was extracted from the ChEMBL database (version 28) using simple text searches, resulting in a set of 557 fragments (including the ones in Figure 6), as obtained from 1059 compounds. The lead series compounds were obtained by a substructure search using the (imidazo(1,2-*a*)pyridine) central scaffold of compound **1** on the surechembl website (https://www.surechembl.org/search) and yielded 138 close analogs, which is a typical number to what a drug hunting program would have access to. The data sets are available online (https://github.com/giovanni-bolcato/deepFMPOv3D). The calculation for this data set requires 8 h on an i9-9820x CPU, using 20 cores.

**Figure 8:**
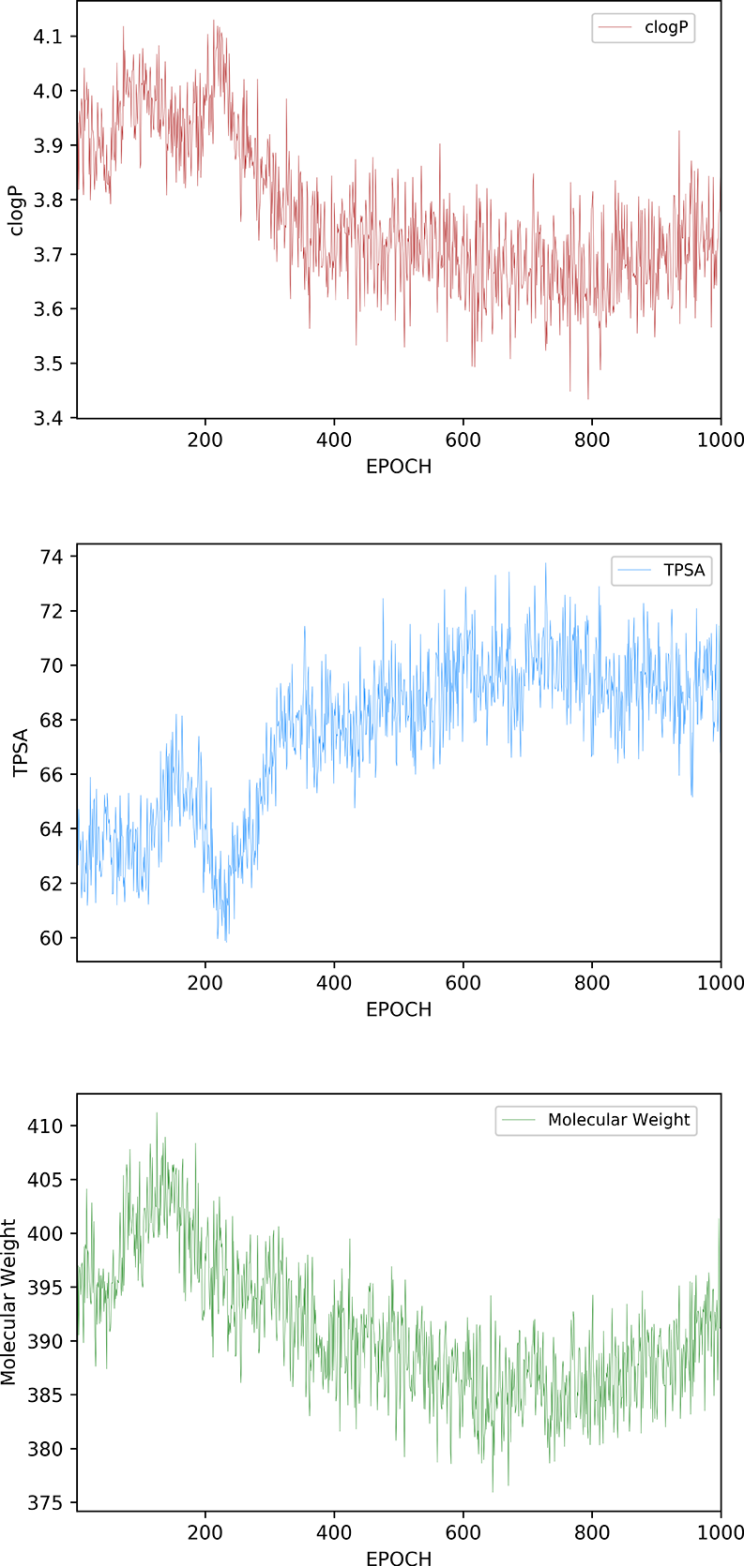
Graphs showing how molecular weight, clogP, and TPSA values change during the epochs, as the mean value of all the compounds for each epoch.

**Figure 9:**
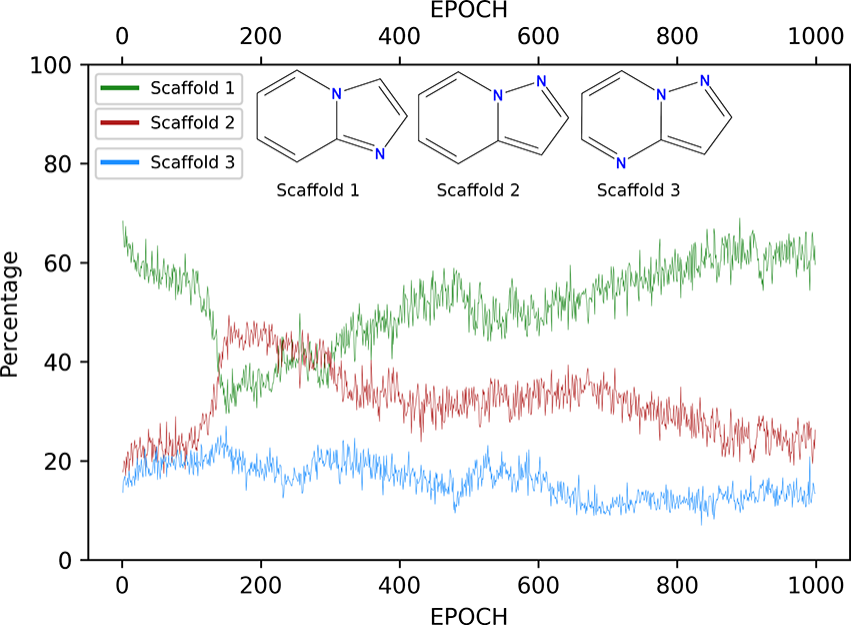
Graph showing the frequency of occurrence of compounds including the central fragment of compounds **1**, **2**, and **3**. The *y*-axis represents the total number of compounds for each epoch (percentage).

DeepFMPO with the ESP-Sim measure generated a total of 6359 unique molecules, when terminated at 1000 epochs. About two-thirds of those were sweet spot compounds. Hence, the agent generated compounds that have all three properties within the desired ranges. This number (ca. 4000) is lower than when using a standard generative method facilitating the selection process and a result of intentional biasing using 3D similarity. The evolution of the percentage of generated molecules that demonstrate properties within the target ranges during the training process is shown in Figure 8, displaying evidence of learning. A significant number of the generated compounds include the central scaffold of compound **2**, and a number of those show a nearly identical substitution pattern to compound **1**. These bioisosteric compounds were observed in early epochs. Another nine-membered scaffold (**3**) was also represented among the generated compounds, see Figure 9. When performing the same experiment but with simpler standard similarity measures (Morgan fingerprints, MACCS keys, and topological fingerprints), no compounds with the central scaffold of compound **2** (or **3**) were generated. This provides an incentive for the use of DeepFMPO with ESP-Sim in scaffold-hopping exercises.

## Discussion

In the current work, we set out to explore the use of a sophisticated similarity metric in generative methods. The power of rewarding compounds that are similar in 3D aspects, in addition to other molecular property constraints, is often underappreciated. It is a challenging task due to the issues involved with conformer generation and molecular alignments. Nonetheless, this is a design strategy that we believe should be given more attention and we discuss why below.

### Molecular Representations in Deep Generative Methods

Deep generative models typically use non-3D methods for representing molecules. Text-based methods and the use of SMILES strings are still the most prevalent representation. The reason for this is probably because SMILES can be massively expressive and that it is trivial to manipulate and transform strings. However, there are some drawbacks with using SMILES strings.^11,64^ A significant problem is that a conservative change can have a huge change in the 3D structure of a molecule. This is important since all molecules are 3D objects. Here, we have addressed this issue by extending the fragment-based DeepFMPO method, where molecules are built from similar fragments, instead of sequences of letters (as is the case for SMILES-based methods). Fragment-based methods are considered intuitive and often mimic the way that medicinal chemists think and design. The approach was recently described by Meyers *et al.* as a method “offering an appealing compromise between molecular expressivity and practicality”.^64^ Hence, a common medicinal chemistry design strategy is to work on molecular series, swapping fragments and substituents in one part of the molecule while keeping other parts of the compounds unaltered. This is often a challenge for generative methods working on SMILES strings,^64,65^ leading us to the next topic of discussion.

### Deep Generative Methods Can Generate Many Compounds

Most generative AI methods produce tens of thousands of unique and diverse high-scoring compounds when used without stringent filters. This is related to Brenner’s underdetermined inverse problem stating that available data does not uniquely specify systems.^66^ Also, although there may be nothing chemically wrong with AI-generated molecules (i.e., all atoms in common valences and charge states), some can be exotic,^67^ and an experienced medicinal chemist would reject them upfront. The issue of such unwanted molecules is manageable from a technical perspective. For example, one can enforce substructure rules and penalize the existence of undesired moieties (e.g., radicals, peroxides, anhydrides, and strained and chemically unstable systems) in the reward functions, or as post-filters.

A more difficult problem to address is how to prune down the numerous generated compounds to the few worth making. In reinforcement learning, a scoring function is used for this purpose. A complicating factor here is that drug discovery is complex and not all factors used in decision-making are easy to capture, and thus, they are not readily converted into rules that the AI methods can use in their reward system. For example, a compound with several stereocenters is usually difficult to make (and resolve) and should consequently get a low reward score unless its building blocks are already available on the shelf. Also, absorption is a critical parameter for the optimization of oral drugs. Permeability over Caco-2 cells is often used as a surrogate when assessing absorption. A complicating factor here is that the uptake over the Caco-2 cells can be hampered by efflux, and in the case of high cell permeation, the efflux is less relevant. A reward function handling such scenarios would require several “if-then-else” statements. They can be included in reward scores but are not always trivial to define and set up for edge cases. In addition, multiparameter optimization becomes increasingly challenging when there are many constraints to fulfill.^68^ In brief, the biggest challenge of deep generative methods is to define relevant reward scores, and this is unfortunately less studied.

Simple drug-likeness rules, multivariate methods for DMPK properties (solubility, permeability, clearance, etc.) and safety, and docking scores are typically included in reward scores as filters. However, several thousands of compounds will inevitably still pass those filters. This is related to the common lack of sufficient high-quality data and the fact that we still often struggle with making predictions to the required accuracy. Prediction of biological activity is an extremely hard problem since many phenomena involved are difficult to quantify precisely. Standard docking scores are most often not sufficient. Although, at times, methods such as free-energy perturbation (FEP) can improve the scoring accuracy for small perturbations of one structure into another but not for major structural changes.^69^ The use of FEP combined with active learning is gaining traction and is showing promise.^70,71^ Nonetheless, when the output contains many structurally diverse molecules, as frequently is the case for expressive SMILES-based generative methods, current methods’ accuracies are not sufficient to filter down many compounds to a selected few. Despite the increasing prevalence of physics-based models in generative modeling, bioaffinity prediction remains very challenging.

Here, we propose shape and electrostatic potential matching as a strategy to bias generative models to propose compounds with different fragments (that are likely bioisosteres) of known lead compounds. The tool is designed to generate novel molecules with optimized properties. One example usage is scaffold hopping. Here, it should be noted that there are many other scaffold-hopping tools available,^72^ ranging from CAVEAT,^73^ which is one of the early 3D database searching programs, to the more recent BROOD.^74^ In the context of generative methods, Langevin *et al.* recently described a new RNN-based algorithm, named SAMOA (scaffold constrained molecular generation), to perform scaffold-constrained molecular design.^65^ Generative methods benefit from the associated reinforcement learning methods, allowing multiobjective molecular design optimization while only exploring the relevant chemical space.

### Using Similarity as a Design Strategy

As mentioned above, current generative AI methods generally suffer from the lack of prediction accuracy. Thus, learning from past drug hunting experiences, we deliberately bias the AI method to generate compounds that are similar to active molecules already discovered. We approach this problem by relying on the similarity principle,^75^ which states that similar molecules tend to have similar properties.^76^ Some advantages to this approach are discussed below. First, by generating molecules similar to the initial set available in the project, confidence in the predictions can be high because they remain in the applicability domain of the model. This is contrary to expressive methods that are designed to fully explore the chemical space and generate structurally diverse compounds, which are consequently also the most uncertain to predict. Second, for similar compounds, the same chemical intermediates and established synthetic routes can often be reused, facilitating speedy progress. Third, sometimes, certain structural fragments (e.g., “privileged structures”^77^) are difficult to replace without severe drops in potency due to specific ligand–protein interactions.

**Figure 10:**
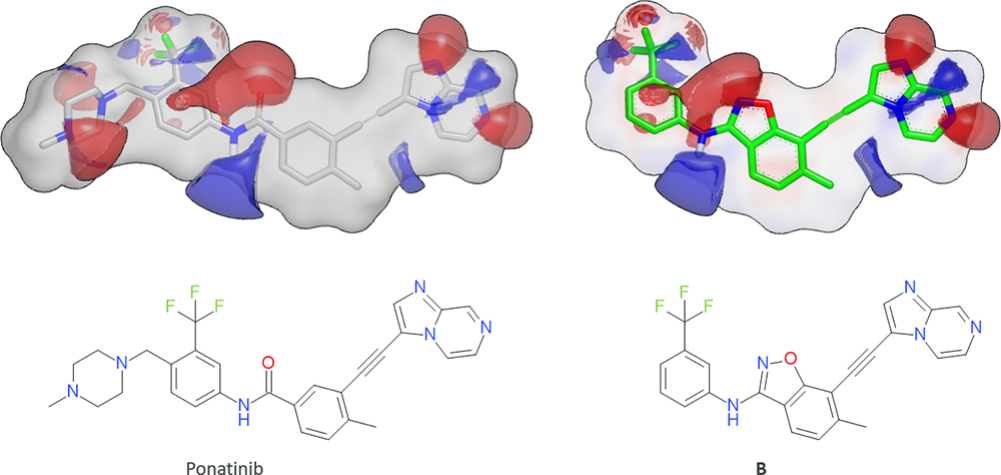
Designing similar compounds can be a good tactic in drug discovery. Here, illustrated with two potent DDR1 kinase inhibitors, the AI-generated compound **B** by Zhavoronkov *et al.* and ponatinib, a marketed multikinase inhibitor, are shown.^78^ The compounds share a rather large common substructure. The hydrogen-bond acceptor and donor functionalities are visualized with electrostatic contours (red: negative, blue: positive). The ESP-Sim Tanimoto value is 0.81 for this pair.

As a related example, the strategy of molecular optimization using similarity was recently applied by Zhavoronkov and coworkers. They reported that deep learning enabled rapid identification of potent DDR1 kinase inhibitors.^78^ Walters and Murcko analyzed the Zhavoronkov *et al.* study and reported that the AI-generated compound **B** (Figure 9) shared a common substructure with an already marketed multikinase inhibitor (ponatinib, Figure 9), which was indeed included in the training set.^79^ In some more detail, they ring-closed a benzamide carbonyl into an isoxazole moiety to yield an equipotent and unique compound.^78^ These two compounds are very similar with regard to shape and electrostatics, see Figure 10. Thus, Zhavoronkov’s AI method successfully mimicked typical medicinal chemistry behavior, keeping certain parts fixed and making minor modifications to others.

It is sometimes believed that computer-aided design (CAD) methods need to provide radically “nonintuitive” different compounds to merit their use. However, believing that CAD approaches should surprise us and produce results that we would not have expected is a tall order. In this context, the scoring functions used in generative methods for reinforcement learning are not designed to extrapolate and do not account for all aspects involved in the drug design process. Thus, the power of current AIs lies more in pattern recognition than in creative discovery.

Palazzesi and Pozzan recently reported a list of over 100 deep generative methods published in the literature between 2017 and 2020.^80^ The methods are innovative and perform well in benchmark studies that measure the models’ ability to, for example, reproduce property distributions and generate valid, diverse, and novel molecules.^81^ One may thus conclude that generative modeling is essentially a solved problem—–given a reward function, we now have the methods for generating molecules that satisfy it. Despite this success, biology and drug discovery remain immensely complex, and it is our viewpoint that current generative methods best serve to augment drug design. To take the next step (full autonomy), calculated predictions need ultrahigh accuracy, and for that, we need to develop a broader understanding of human biology. The state of AI in drug design may be seen as analogous to the automotive industry. While the future of autonomous vehicles is promising and exciting, we are not near fully autonomous cars yet. Candidate drugs, as well as cars, still require human attention, given the complexity involved and the vast amount of edge cases that are nontrivial to code up efficiently. Thus, humans (with domain knowledge) are still very much needed in the process, to steer the tools and triage the output. In this context, we would like to highlight the Gruenif.ai tool where the user can provide feedback interactively while molecules are generated.^82^ Such “human-in-the-loop” methods can be very effective. Future versions of DeepFMPO will include such functionalities.

## Conclusions

The use of sophisticated computational methods for *de novo* design is attractive, and deep generative methods have gained a lot of attention. Significant progress has been made when it comes to generating molecules. However, scoring them accurately remains a major challenge. Real-life project experience informs us that *in silico* predictions (e.g., synthesis, potency, and properties) are constantly improving, but they are generally not accurate enough to prioritize a handful of compounds for synthesis from a long list of high-scoring AI-generated molecules. Thus, what really needs solving is being able to do ultraaccurate predictions to advance the field to the next level. Until then, the approach of biasing molecular design toward compounds similar to known actives will remain as one pragmatic and fruitful way to success.

Here, we present a 3D fragment-based reinforcement learning approach for the generation of novel molecules with optimized properties, called “DeepFMPO v3D”. We furthermore developed a python package, ESP-Sim, for calculating molecular shape and electrostatic similarities. We benchmarked ESP-Sim on a variety of tasks including the evaluation of detailed 3D similarities, protein–ligand docking, and rescoring of docked ligands and reported competitive performances. The inclusion of ESP scores into DeepFMPO promotes the generation of compounds similar to existing lead molecules, toward desirable sweet spot properties. The proposed method allows the calculation of high-quality partial charges (e.g., RESP with B3LYP/6-31G**) obtained using the quantum chemistry program Psi4. In a scaffold-hopping case study, we show that our approach of using shape and electrostatic similarities performs well. DeepFMPO v3D ranks known equipotent scaffolds higher and generates them earlier (i.e., speedier). The way that the 3D method is implemented makes the approach essentially alignment-independent (on a molecular level) and does not require knowing of the bioactive conformation. Both DeepFMPO v3D and ESP-Sim are freely available online.

## Supporting Information Available

Comparison of electrostatic similarities via different charge distributions evaluated with Carbo similarity scores; comparison of ESP-Sim to EON scores; benchmark of ESP-Sim scores on their ability to identify ligands for the D4 dopamine receptor and benchmark of ESP-Sim scores on DUD-E; distribution of ESP-Sim scores compared to 2D fingerprint similarities; code to reproduce the MCS Tanimoto score in Table 2 (PDF)

## Notes

The ESP-SIM code can be found here: https://github.com/hesther/espsim. The repository also contains the data sets (3000 molecules with QM RESP charges, 451 molecules from DeepFMPO, and instructions to download D4 and DUD-E) and scripts to reproduce all benchmarks. The DeepFMPO_v3D code version together with the associated data sets used in this study are here: https://github.com/giovanni-bolcato/deepFMPOv3D.
