# Supplementary material for: On the Value of Using 3D Shape and Electrostatic Similarities in Deep Generative Methods
Source: J Chem Inf Model. 2022 Mar 10;62(6):1388–98. doi: 10.1021/acs.jcim.1c01535 (PMC8965872; doi:10.1021/acs.jcim.1c01535)
Supplement: Supplementary file 1 — ci1c01535_si_001.pdf [file ci1c01535_si_001.pdf]

Supporting information

"On the value of using 3D-shape and electrostatic similarities in deep generative methods"

Giovanni Bolcato[a], Esther Heid[b] and Jonas Boström*[,c]

[a]Molecular Modeling Section, University of Padova, 35131 Padova, Italy. [b]Department of Chemical Engineering, Massachusetts Institute of Technology, Cambridge, 02139 Massachusetts, United States [c]Medicinal Chemistry, Early CVRM, BioPharmaceuticals R&D, AstraZeneca, 431 50 Mölndal, Sweden.

Corresponding Author

* Telephone number: +46 73 7065251. E-mail: dr.jonas.bostrom@gmail.com

1. ESP-Sim benchmark study 1: Influence of partial charges

Fig. S1 depicts the electrostatic potential similarities between molecules with RESP vs other partial charges via the Carbo similarity (an analogous figure for Tanimoto similarity is given in the main article). Code to reproduce this benchmark is available on GitHub (https://github.com/hesther/espsim).

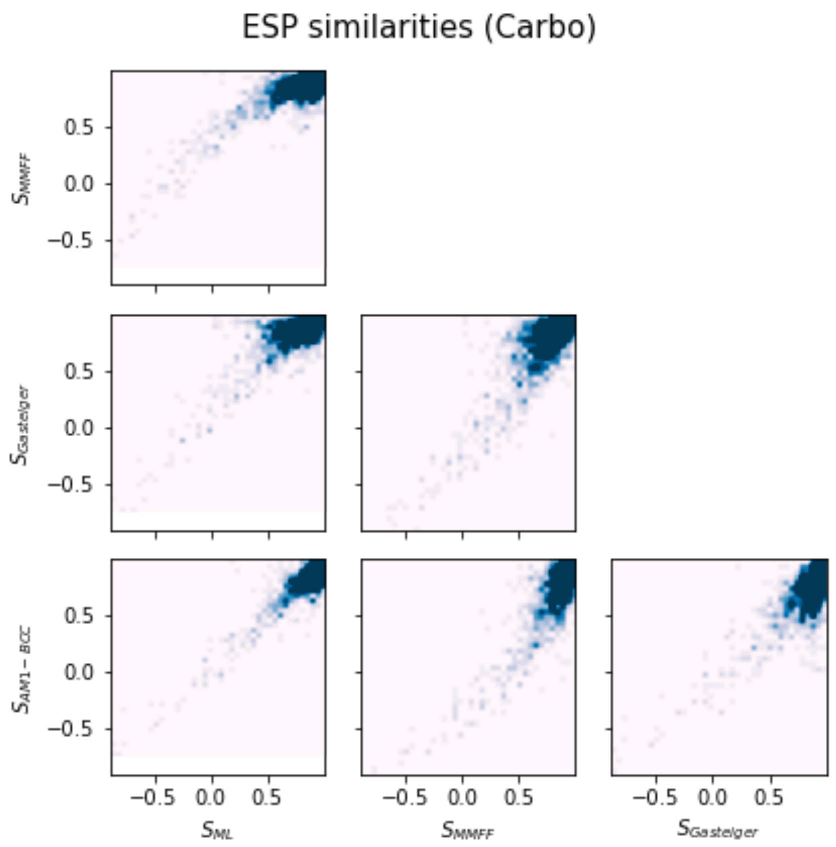

Fig. S1: Electrostatic potential similarities between molecules with RESP partial charges to molecules with Gasteiger, MMFF, ML or AM1-BCC partial charges (at the exact same geometries) via Carbo similarity.

2. ESP-Sim benchmark study 2: Comparison to EON

Similarities for about 450 fragments from DeepFMPO were computed via EON (ETanimoto, dielectric of 1, inner mask of 0.05, outer mask of 0.0005, salt concentration of 0.04) with AM1-BCC, Gasteiger and MMFF charges, and compared to the respective ESP-Sim Tanimoto scores. To this aim, 100 molecules were randomly chosen as reference and a single conformation was created with RDKit's *EmbedMolecule* function. For each of the remaining molecules, ten conformations were created with RDKit's *EmbedMultipleConfs* function and aligned using RDKit's *Align* function using Crippen logP atom contributions [1]. Only the conformation yielding the best alignment score was kept, and the aligned conformation used as input for both EON and ESP-Sim. Code to reproduce this benchmark is available on GitHub (https://github.com/hesther/espsim). The computation of ESP-Sim similarities of all pre-aligned pairs with pre-computed charges (about 100,000 pairs) took 35s on a 2.2 GHz Quad-Core Intel Core i7 MacBook Pro laptop on a single core, which corresponds to 0.35ms per pair on average.

Table S1 summarizes the mean absolute deviations of the observed similarities S, as well as the Spearman correlation between the EON and ESP-Sim similarities averaged over the 100 reference molecules. Fig. S2 depicts a direct comparison of the observed scores, where we find satisfactory agreement across all charge distributions.

Table S1: Mean absolute deviations of the similarity scores obtained via ESP-Sim from scores obtained via EON, as well as Spearman correlations between the observed scores for different charge distributions accessible via EON.

Partial charges	MAE of S	Spearman correlation
**Gasteiger**	0.12	0.83
**MMFF**	0.09	0.81
**AM1-BCC**	0.11	0.75

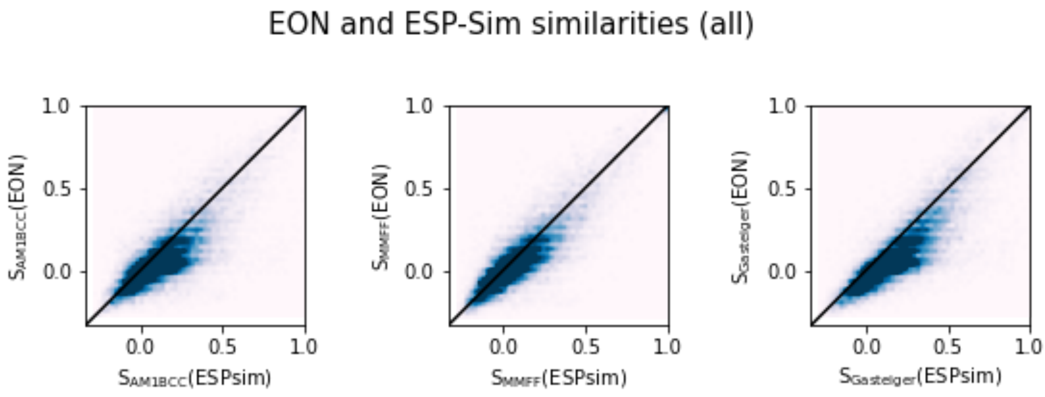

Fig. S2: Comparison of similarity scores obtained via EON and ESP-Sim for different charge distributions.

3. ESP-Sim benchmark study 3: D4 dopamine receptor

To evaluate the ability of ESP-Sim scores to highly rank active ligands over a set of inactivate decoys, a recently published experimental screening study for the D4 dopamine receptor was employed [2]. In their study, Lyu et al tested 549 compounds for interactions with D4 in vitro, thus creating a set of experimentally verified actives and inactives towards this target. ESP-Sim performance on re-ranking the 549 compounds (which are recorded in different poses, thus summing to 4933 which are used in the following) was compared against a recent benchmark from GitHub, https://github.com/ljmartin/d4-rescore, of a set of re-scoring functions. To obtain ESP-Sim scores, each of the 4933 sdf files was transformed into a RDKit molecule and randomly embedded in 10 different conformations using RDKit's *EmbedMultipleConfs* function and aligned via RDKit's *Align* function using Crippen logP atom contributions [1]. Only the conformation leading to the best alignment score to a known ligand in its co-crystallized structure was kept and its electrostatic and shape similarity to the ligand computed. Code to reproduce this benchmark is available on GitHub (https://github.com/hesther/espsim). The embedding, aligning and computation of ESP-Sim similarities took 825s on 4 cores of a 2.2 GHz Quad-Core Intel Core i7 MacBook Pro laptop. Only aligning and scoring amounted to 34s, whereas only scoring pre-computed, pre-aligned poses took 14s (2.8ms per pose, for three different charge distributions per pose).

Results are given in Table S2. While most re-scoring functions do not perform better than random ranking, although they take into account the docked ligand conformation and the protein crystal structure, ESP-Sim scores (sum of ESP and shape similarity) yield good performances across different partial charge distributions (see Table S2), as well as different similarity metrics and combinations of the shape and ESP scores (data not shown). ESP-Sim thus provides an easy, computationally inexpensive and open-source framework for re-scoring of protein-ligand interactions in the case of the D4 dopamine receptor. In the following, we aim to generalize this observation by benchmarking on a larger dataset, namely the 102 targets from DUD-E.

Table S2: Comparison of performance of docking, rescoring and ligand-centric approaches on identifying ligands for the D4 dopamine receptor. We report the area under the curve (AUC), of the receiver-operator characteristic (ROC), area under the curve after a semilog transformation of the ROC (LogAUC) adjusted by the LogAUC of the random curve (0.1446) and Boltzmann-enhanced discrimination of ROC (BEDROC). Data from https://github.com/ljmartin/d4-rescore.

	AUC	Adj. LogAUC	BEDROC
**ESP-Sim Tanimoto ML (this work)**	0.626	0.075	0.432
**ESP-Sim Tanimoto MMFF (this work)**	0.640	0.080	0.422
**ESP-Sim Tanimoto Gasteiger (this work)**	0.614	0.065	0.403
**Smina Score**	0.308	0.029	0.308
**RFScore**	0.423	-0.028	0.186
**PLECScore**	0.584	0.034	0.281
**NNSCore**	0.509	0.001	0.224
**Feature Map Vector Score**	0.649	0.089	0.439
**RF Score VS**	0.506	-0.003	0.180

4. ESP-Sim benchmark study

To benchmark the ability of ESP-Sim to prioritize active against decoy molecules for different target proteins, ESP-Sim scores (sum of ESP similarity and shape similarity) were computed on the 102 targets of DUD-E. Each compound was embedded in ten conformations using RDKit and scored against a known ligand in its co-crystallized conformation. RDKit's *EmbedMultipleConfs* function was used to create conformers, and aligned with the known ligand using RDKit's *Align* function using Crippen logP atom contributions [1]. Tanimoto shape scores were computed for each aligned conformer, where the highest scoring conformation was kept. The electrostatic similarity to the ligand was then computed only on this conformer. Code to reproduce this benchmark is available on GitHub (https://github.com/hesther/espsim). Embedding all molecules (about 1.5 million) took 9.4h (23ms per molecule) on 40 cores of an Intel Xeon Platinum 8260 computer, whereas computing ESP-Sim scores took 1.8h (4.6ms per molecule). We note that the use of faster embedding routines beyond RDKit could significantly decrease computation time.

Results are reported in Table S3 and compared against a variety of other ligand-based approaches as reported in Ref. [2] and [3]. ESP-Sim outperforms most other approaches, with the notable exceptions of eSim [3] and mRAISE [4]. The eSim method takes into account electrostatic potential, shape and hydrogen donor/acceptor similarities, where especially the latter is important for good performance [2]. Without hydrogen donor/acceptor similarities, *i.e.* taking into account only electrostatics and shape, ESP-Sim outperforms eSim when using the ML or MMFF partial charge model. We furthermore note that the ML partial charge model leads to the best performances of ESP-Sim, although the differences are rather small. ESP-Sim also outperforms ROCS for both shape-only and shape + chemistry-based scoring (where the 'color' features describe hydrogen donor, hydrogen acceptor, charged, and hydrophobic functional groups, as well as rings). ESP-Sim therefore shows satisfactory performance in the upper quarter of available algorithms, albeit it is outperformed by eSim [3] and mRAISE [4].

We note that many docking-based approaches outperform all ligand-based methods reported in Table S3 on the DUD-E targets, including ESP-Sim. However, ESP-Sim uses less information, is fast, easy to use and open-source, and requires

only a few conformations per compound. We therefore conclude that ESP-Sim is a valid and fast alternative to currently employed ligand-based re-scoring approaches.

Table S3: Comparison of performance of ligand-centric methods on the 102 DUD-E targets. Evaluated metrics: Area under the curve (AUC), of the receiver-operator characteristic (ROC), area under the curve after a semilog transformation of the ROC (LogAUC) adjusted by the LogAUC of the random curve (0.1446), Boltzmann-enhanced discrimination of ROC (BEDROC), ROC-based enrichment factor at 1% of decoys (EF1). Data from Ref. [3] and [4].

	AUC	Adj. LogAUC	BEDROC	EF1
**ESP-Sim Tanimoto ML (this work)**	0.700	0.191	0.289	13.9
**ESP-Sim Tanimoto MMFF (this work)**	0.675	0.179	0.276	14.6
**ESP-Sim Tanimoto Gasteiger (this work)**	0.671	0.172	0.268	14.0
**mRAISE**	0.740			23.5
**eSim -pscreen full**	0.755			
**eSim -pscreen only Shape+Coulomb**	0.672			
**ROCS Color**	0.663			
**ROCS Shape**	0.596			
**WEGA**	0.564			
**OptiPharm (robust)**	0.560			
**VAMS**	0.560			
**USR**	0.554			
**USR (shape)**	0.520			

5. ESP-Sim Tanimoto distribution

Tanimoto distributions of calculated similarities for several methods (MACCS Keys, Morgan FPs (radius=2), Atom-Pairs and ESP-SIM) were calculated and are depicted in Fig. S3. The similarity values were calculated for all pairs of fragments used in the present work.

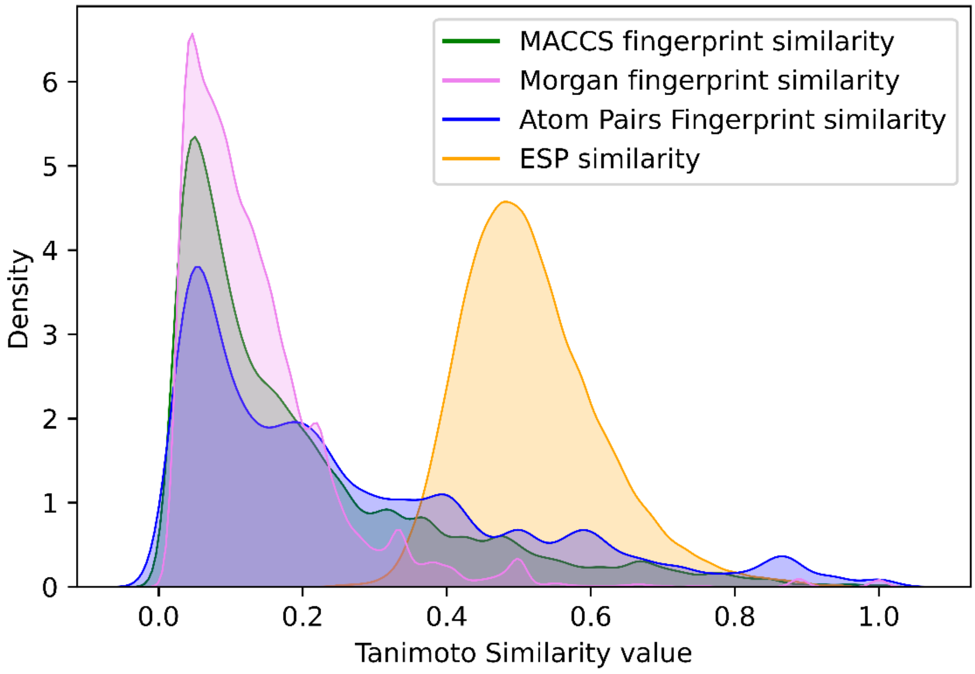

Fig. S3: Distribution of ESP similarity scores (average of electrostatic and shape similarities).

6. MCS-Tanimoto equation

An example of how the MCS-Tanimoto in Table 2 is computed is shown below:

```
>>> import rdkit
>>> from rdkit import Chem
>>> from rdkit.Chem import rdFMCS
>>> def calcMCStanimoto(ref_mol,target_mol):
...     numAtomsRefCpd = float(ref_mol.GetNumAtoms())
...     numAtomsTargetCpd = float(target_mol.GetNumAtoms())
...     pair_of_molecules = [ref_mol, target_mol]
...     numCommonAtoms =
rdFMCS.FindMCS(pair_of_molecules,atomCompare=rdFMCS.AtomCompare.CompareElements,bondCompare=rd
FMCS.BondCompare.CompareOrderExact,matchValences=True).numAtoms
...     mcsTanimoto = numCommonAtoms/((numAtomsTargetCpd+numAtomsRefCpd)-numCommonAtoms)
...     return mcsTanimoto
...
>>> target_smi = 'Cc1cc(C)n2ncc(C)c2c1'
>>> ref_smi = 'Cc1cc(C)c2ncc(C)n2c1'
>>> ref_mol = Chem.MolFromSmiles(ref_smi)
>>> target_mol = Chem.MolFromSmiles(target_smi)
>>> print("MCS Tanimoto: %s" % calcMCStanimoto(ref_mol,target_mol))
MCS Tanimoto: 0.5
```

References

[1] Wildman, S. A., and Crippen, G. M. J. Prediction of physicochemical parameters by atomic contributions. *Chem. Inf. Comput. Sci.*, 1999, 39, 868-873

[2] Lyu, J., Wang, S., Balius, T. E., Singh, I., Levit, A., Moroz, Y. S., O'Meara, M. J., Che, T., Algaa, E., Tolmachova, K., Tolmachev, A. A., Shoichet B. K., Roth, B. L. and Irwin, J. J. Ultralarge library docking for discovering new chemotypes. *Nature*, 2019, 566(7743), 224-229.

[3] Cleves, A. E., Johnson, S. R., and Jain, A. N. Electrostatic-field and surface-shape similarity for virtual screening and pose prediction. *J. Comput. Aided Mol. Des.*, 2019, 33(10), 865-886

[4] von Behren, M. M., Bietz, S., Nittinger, E., and Rarey, M. mRAISE: An alternative algorithmic approach to ligand-based virtual screening. *J. Comput. Aided Mol. Des.*, 2016, 30(8), 583-594.
